# Supplementary material for: Unveiling Molecular Mechanisms of Nitric Oxide-Induced Low-Temperature Tolerance in Cucumber by Transcriptome Profiling
Source: Int J Mol Sci. 2022 May 17;23(10):5615. doi: 10.3390/ijms23105615 (PMC9146554; doi:10.3390/ijms23105615)
Supplement: Supplementary file 1 [file ijms-23-05615-s001.zip › ijms-1720261-supplementary.pdf]

Abbreviations:

ABA, abscisic acid; BP, biological process; BR, brassinosteroid; Ca^{2+}, calcium; CC, cellular component; CHI, chalcone isomerase; CHS, chalcone synthase; CI , Chilling damage index; CI, chilling damage index; CTR1, constitutive tripleresponse 1; DEGs, differentially expressed genes; EBF1_2, EIN3-binding F-Box 1_2; EIN2 CEND, carboxyl end of EIN2 protein; EIN2, ethylene-insensitive protein 2; Ein3, ethylene-insensitive protein 3; ERF1, ethylene-responsive f,actor1; ETH, ethylene; ETR, ethylene receptor; F3H, 3-hydroxylase; Fv/Fm, maximum photochemical efficiency; GA, gibberellin; GA3oX, gibberellin 3 beta-dioxygenase; GO, Gene Ontology; GSH, glutathione; GSTs, glutathione S-transferases; H_2S, hydrogen sulfide; IAA, auxin; JA, jasmonic acid; KEGG, Kyoto Encyclopedia of Genes and Genomes; LHCA, light-harvesting complex I chlorophyll a/b binding protein, LHCB, light-harvesting complex II chlorophyll a/b binding protein; LT, low temperature; MCM, DNA replication licensing factor; MDA, malondehyde; MF, molecular function; NO, nitric oxide; NPR1, regulatory protein; PAL, phenylalanine ammonialyase; PCA, principal component analysis; PPFD, photosynthetic photon flux density; qRT-PCR, quantitative real-time PCR; RNA-Seq, RNA sequencing; ROS, oxygen species; SA, salicylic acid; SA, salicylic acid; TBARS, thiobarbituric acid reactive substances; t-CA, trans-cinnamic acid; TFs, transcription factors.

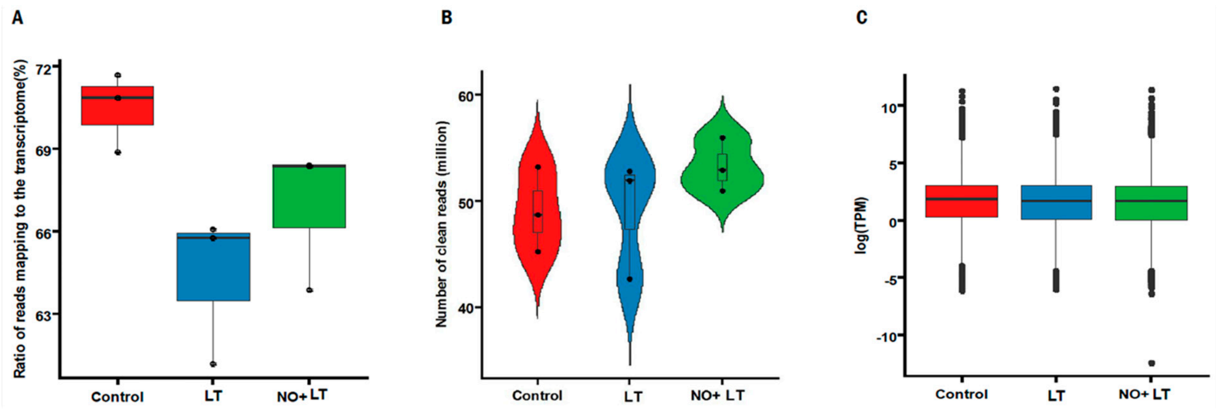

Figure S1. A, the ratio of reads mapping to the transcriptome of each treatment in cucumber plants; B, number of clean reads of each treatment n cucumber plants; and C, Log(TPM) of each treatment in cucumber plants.

Table S1. The primer sequences for qRT-PCR

Gene ID	Forward primer	Reverse primer
Actin (*CsaV3_6G041900.1*)	5′-ATGTGCCTGCTATGTATGTTG-3′	5′-GCTCCGATGGTGATGACTT-3′
WRKY (*CsaV3_2G013650.1*)	5′-TTGAGTATGGATGGCAGCGT-3′	5′-GCTCCCTTCTTCTCCTCTAGC-3′
MYB (*CsaV3_7G004040.1*)	5′-ACAAAATTACCCGGACGAACC-3′	5′-CTCCTCCTCATGCTTCTTTGC-3′
HD-ZIP (*CsaV3_6G045240.1*)	5′-TCAGTCGATGTTGGATGGCT-3′	5′-TTTCTCTCCGGTTCGAGCTT-3′
b-ZIP (*CsaV3_3G037220.1*)	5′-GGATGAGGTTTGGTCCGAGA-3′	5′-GTGTGGCAGATTCCGGATTC-3′
Peroxidase (*CsaV3_5G005000.1*)	5′-TTCTCCAAAGCACTGCCCTA-3′	5′-ACAATGGAGGGTAGGTTGGG-3′
MCM5 (*CsaV3_2G003500.1*)	5′-GTCAAGGACCAAGGCAAAGG-3′	5′-TGGTTCTTCTCCAGGCTGAG-3′
MCM6 (*CsaV3_6G053180.1*)	5′-ACATCAGGGATCAGGTTGCA-3′	5′-TAGTGTGGCCTGTATTCCGG-3′
PAL (*CsaV3_6G039690.1*)	5′-GGCAGCAATTATGGAGCACA-3′	5′-AGGCTTTTGGAGGGGATCAA-3′
NPR1 (*CsaV3_3G040450.1*)	5′-ATCTTGGCGGCTAGGAGTTT-3′	5′-TACTACTCCCTGTCGACGGA-3′
LHCA1 (*CsaV3_5G025740.1*)	5′-GGAGAAGTGCCAGAGAACCT-3′	5′-GCTTTAACCCAGTTGCCCAA-3′
LHCB1 (*CsaV3_6G051520.1*)	5′-GTTTGGTGAAGCTGTGTGGT-3′	5′-CCCATCAACACAACTTGGCA-3′
GA3ox (*CsaV3_7G032870.1*)	5′-CATAAACTCAAGGCCGCTCG-3′	5′-ATGAGGCCAGAGTTTTCGGA-3′
PR1 (*CsaV3_7G007620.1*)	5′-GGACCTGTCAGTTGGGATGA-3′	5′-CCCATATGGCCCGTTAGAGT-3′
ERF1 (*CsaV3_3G012170.1*)	5′-CCTAATCTCCGAGGGCACAT-3′	5′-GGCGGCGAACTCCTCTATAA-3′

Table S2. Data description of RNA-Seq reads for the three cucumber samples with three replicates

samples	Total reads (M)	Total bases (G)	Mapping ratio	Clean total reads (M)	Clean total bases (G)	Q30 bases
Control 1	48.72	7.31	71.67	48.56	7.25	0.93
Control 2	45.28	6.79	70.85	45.14	6.75	0.93
Control 3	53.18	7.89	68.88	53	7.92	0.93
LT1	42.68	6.4	61.18	42.51	6.36	0.93
LT2	52.75	7.91	65.75	52.59	7.86	0.93
LT3	51.94	7.79	66.06	51.74	7.73	0.93
NO + LT 1	51.02	7.65	63.85	50.84	7.6	0.93
NO + LT 2	55.94	8.39	68.41	55.74	8.34	0.93
NO + LT 3	52.85	7.93	68.35	52.72	7.88	0.94
